# Evaluation of the antibacterial activity of selected Pakistani honeys against multi-drug resistant *Salmonella typhi*

**DOI:** 10.1186/s12906-015-0549-z

**Published:** 2015-02-26

**Authors:** Muhammad Barkaat Hussain, Abdul Hannan, Naeem Akhtar, Ghulam Qadir Fayyaz, Muhammad Imran, Sidrah Saleem, Imtiaz Ahmed Qureshi

**Affiliations:** Department of Microbiology, Faculty of Medicine, King Abdul Aziz University, Rabigh Branch, 21589 Saudi Arabia; Department of Microbiology, University of Health Sciences Lahore, Khayaban-e-Jamia Punjab, Lahore, 54600 Pakistan; Department of Pathology, Rawalpindi Medical College, Rawalpindi, Pakistan; Department of Plastic Surgery, Services Institute of Medical Sciences, Lahore, Pakistan; Faculty of Medicine, King Abdul Aziz University, Rabigh Branch, 21589 Saudi Arabia; Department of Pathology, Faculty of Medicine, King Abdul Aziz University, Rabigh Branch, 21589 Saudi Arabia

**Keywords:** Honey, Typhoid fever, *Salmonella typhi*, Antibacterial activity, Phenol equivalence, Inhibine antibacterial units

## Abstract

**Background:**

The development of resistance to conventional anti-typhoid drugs and the recent emergence of fluoroquinolone resistance have made it very difficult and expensive to treat typhoid fever. As the therapeutic strategies become even more limited, it is imperative to investigate non-conventional modalities. In this context, honey is a potential candidate for combating antimicrobial resistance because it contains a broad repertoire of antibacterial compounds which act synergistically at multiple sites, thus making it less likely that the bacteria will become resistant. The in vitro antibacterial activity of 100 unifloral honey samples against a blood culture isolate of multi-drug resistant (MDR) *Salmonella typhi* were investigated.

**Methods:**

All honey samples were evaluated for both total (acidity, osmolarity, hydrogen peroxide and non-peroxide activity) and plant derived non-peroxide antibacterial activity by agar well diffusion assay at 50% and 25% dilution in sterile distilled water and 25% in catalase solution. Manuka (Unique Manuka Factor-21) honey was used for comparison. The phenol equivalence of each honey sample from 2% to 7% (w/v) phenol was obtained from regression analysis. The antibacterial potential of each honey sample was expressed as its equivalent phenol concentration. The honey samples which showed antibacterial activity equivalent to or greater than manuka honey were considered therapeutically active honeys.

**Results:**

Nineteen honey samples (19%) displayed higher hydrogen peroxide related antibacterial activity (16–20% phenol), which is more than that of manuka honey (21-UMF). A total of 30% of the honey samples demonstrated antibacterial activity between 11 and 15% phenol similar to that of manuka honey while 51% of the honey samples did not exhibit any zone of inhibition against MDR-*S. typhi* at 50% (w/v) dilution. None of the indigenous honey samples displayed non-peroxide antibacterial activity. Only manuka honey showed non-peroxide antibacterial activity at 25% dilution (w/v) in catalase solution.

**Conclusions:**

The honey samples which displayed antibacterial activity equal to or greater than manuka honey may be useful in the clinical conditions where higher hydrogen peroxide related antibacterial activity is required. Manuka honey, which is known to possess non-peroxide antibacterial activity, warrants further evaluation in a suitable typhoid animal model.

## Background

Typhoid fever remains an important health problem in the developing world and is a leading cause of fever in returning travelers [1]. Resistance to the conventional anti-typhoid antibiotics ampicillin, cotrimoxazole and chloramphenicol is common in Asia, including in Pakistan [2]. However, recent emergence of fluoroquinolone resistance in typhoidal salmonellae has further complicated the situation [3]. Resistance to cephalosporins like ceftriaxone has almost exhausted the stock of useful anti-typhoid drugs and there is obvious concern that we may be entering a pre-antibiotic era where typhoid fever becomes untreatable [4]. Moreover, the recent emergence of extended-spectrum ß-lactamases (ESBLs) in *S. typhi* limits the therapeutic options to tigecycline and carbapenems as the last resort [5]. Consequently, efforts are needed to evaluate and develop new agents to combat this ongoing health crisis.

Development of an antibiotic based on a single compound strategy is not effective because of widespread antimicrobial resistance and adverse drug reactions [6]. Therefore, there is an urgent need to explore those antibacterial compounds which are less prone to initiate the development of bacterial resistance and are free from side effects. In this context, honey is a potential candidate for combating antimicrobial resistance because it contains a repertoire of antibacterial compounds which act synergistically at multiple sites, thus minimizing the opportunity for the bacterium to become resistant [7-10]. To date, antibacterial resistance against honey has not been documented.

Moreover, honey blocks quorum sensing [11] and disrupts the formation of biofilms [12-14]. Unlike conventional antibiotics, honey does not disturb the growth of gastric beneficial bacteria, but instead it promotes the growth of bifidobacteria and lactobacilli [15,16]. A variety of prebiotics have also been identified in honey [17]. More recently, a study revealed that honey can prevent the formation of free radicals in an inflammatory colitis model and application of intra-rectal honey is as effective as prednisolone [18]. These peculiar characteristics of honey make it a potential candidate for treatment of drug-resistant bacterial infections, particularly in those involving the gastrointestinal tract. Most early studies focused on the antibacterial activities of honey against bacteria which are implicated in skin infections and therefore, honey has been approved as a drug for the treatment of skin infections and burns [19]. Previously, we evaluated the antibacterial activities of two indigenous honeys, black seed and shain honey, against multi-drug resistant typhoidal salmonellae [20]. Although Pakistanis a key producer of several varieties of honey, most have not been screened for their antibacterial activities. Therefore,100 samples of Pakistani unifloral honey from 19 common species specific floral types were collected from different geographical areas of Pakistan and were screened for both total and residual antibacterial activity against a clinical isolate of MDR-*S. typhi*. Medical grade manuka honey was used for comparison.

## Methods

### Bacterial strain

A blood culture isolate of MDR-*S. typhi* was obtained from the Institute of Microbiology, Military Hospital, Rawalpindi, Pakistan. Biochemical identification of *Salmonella typhi* was reconfirmed by an API-20E (bioMerieux, Inc. France) test kit and serological identification was performed by *Salmonella* O, H and Vi antisera (BD Difco, USA). The isolate was preserved in Microbank vials (Pro-Lab Diagnostics, UK) at −80°C. Before use, the strain was sub-cultured on sheep blood agar and its characteristic features were confirmed.

### Honey samples

A total of 100 honey samples of 19 different botanical origins (flora) from different geographical locations of Pakistan were collected and investigated for antibacterial potential against MDR*S. typhi* (UHS-16) (Table 1). These samples were obtained from commercial apiarists (n = 90), Honey Bee Research Farm, Punjab University (PU), Lahore (n = 4) and Honey Bee Research Farm, National Agricultural Research Council (NARC), Islamabad (n = 6) from April 2006 to December 2009. Honey samples were collected from 21 districts of Pakistan (Table 2). Identification of the plant source of the honey samples were performed on the basis of the geographical location, flowering plants, season, aroma and color of each honey [21,22]. The honey samples were placed in the dark at room temperature (20–30°C).

**Table 1:** **Details of Pakistani unifloral honeys screened for antibacterial activity (n = 100) against MDR-** ***Salmonella typhi*** **(UHS = 16)**

**Serial no**	**Common name**	**Botanical name**	**Code no**	**No. of samples**
1	Acacia/Phulai	*Acacia modesta*	AC	15
2	Beri	*Ziziphus jujuba*	BE	29
3	Bhaiker	*Justicia adhatoda*	BK	05
4	Black locust	*Robinia pseudoacacia*	RPA	03
5	Black seed	*Nigella sativa*	BS	05
6	Clover/Shatala	*Trifolium alexandrium*	CL	04
7	Date palm	*Phoenix dactylifera*	DT	01
8	Eucalyptus	*Eucalyptus spp*.	EU	01
9	Garanda	*Carissa opaca*	GN	04
10	Honey dew	*Honey dew*	HD	01
11	Lavender	*Lavendula intermedia*	LAV	01
12	Litchi	*Litchi chinesis*	LY	02
13	Loquat	*Eriobotrya japonica*	LO	01
14	Mustard	*Brassica spp*.	MH	04
15	Orange blossom	*Citrus sinensis*	CT	12
16	Russian olive	*Elaeagnus angustifolia*	RO	05
17	Shain	*Plectranthus rugosus wall*	SH	01
18	Sunflower	*Helianthus annus*	SN	05
19	Walati kikhar	*Prosopis spp.*	WK	01

**Table 2:** **District (n = 21) wise distribution of Pakistani unifloral honey used in this study**

**S.No**	**District**	**No of samples**
1	Islamabad	15
2	Sargodha	13
3	Attock	09
4	Gilgit	09
5	Rawalpindi	09
6	Lahore	07
7	Karak	06
8	Haripur	05
9	Jhelum	05
10	Bahalwalpur	03
11	Mansehra	03
12	Vehari	03
13	Badeen	02
14	Chakwal	02
15	Mirpur, Azad Kashmir	02
16	Sheikhupura	02
17	Bannu	01
18	Hafizabad	01
19	Mianwali	01
20	Sukhar	01
21	Swaat	01

### Screening of honey

#### Agar well diffusion assay

All honey samples were screened by agar well diffusion assay as adopted from the work of Allen et al., (1991) with slight modifications [21]. Agar well diffusion is the most frequently used assay for determining the antibacterial activity of honey because of its simplicity and low cost [22-24]. Manuka honey (UMF- 21) was used for comparison. Manuka honey has an antibacterial activity equivalent to 21% phenol (w/v), as previously determined against a reference strain of *Staphylococcus aureus* (ATCC 25923) [21] and was kindly provided by Professor Peter C. Molan, Department of Biological Sciences (Honey Research Unit), University of Waikato, New Zealand. Cefixime (5 μg) disc and ciprofloxacin (5 μg) disc, (Oxoid, Basingstoke, UK) were used as positive controls. These antibiotics gave a zone of inhibition which was used to account for day to day variations. Sterile distilled water (SDW) was used as the negative control. As treatment with catalase solution can neutralize the hydrogen peroxide related antibacterial activity in honey, those honey samples which retained antibacterial activity after addition of catalase solution are considered to have plant derived non-peroxide activity [21,22].

MDR-*S. typhi* was sub-cultured onto sheep blood agar plates and incubated for 24 hours at 37°C. Morphologically identical colonies were picked and suspended in sterile 10 ml tryptic soya broth (TSB) and incubated for approximately 5 hours at 37°C to attain a fully logarithmic phase culture. The culture was adjusted to 0.5 McFarland turbidity calculated at 540 nm using sterile TSB as a blank and a diluent with a 1 cm pathway.

To prepare the large square Bio-Assay plates (low profile, sterile polystyrene, 649560–241 × 241 × 20 mm, Nalge Nunc International, USA), 150 ml Mueller Hinton (MH) agar (Oxoid Ltd, UK) was autoclaved and kept at 50°C for 30–35 minutes prior to seeding with 100 μl of *S. typhi* culture adjusted to 0.5 McFarland turbidity. The agar was mixed thoroughly by swirling and poured into a Bio-Assay plate and maintained at 4°C overnight. The next day, 47 wells were made in the agar with a sterile 9 mm cork borer using a quasi-Latin square as a template. The template was constructed on a black card (241 × 241 mm), and a 30 mm grid was drawn on the card. The wells were centered at each of the 47 intersections and each intersection of the grid was numbered using a quasi-Latin square. This allows the samples to be placed randomly on the plate. On two intersections, wells were not made and used for the positive controls, cefixime (5 μg) and ciprofloxacin (5 μg).

#### Sample preparation

Initially a primary solution (50% w/v) from each honey sample was prepared by adding 2 g of well-mixed honey to 2 ml of sterile distilled water (SDW) in universal bottles. They were incubated at 37°C for 30 minutes to aid dissolution by intermittent stirring. Secondary honey solutions (25% w/v) were prepared by taking 1 ml of honey from each primary solution and mixing with 1 ml of SDW or 1 ml catalase solution. The catalase solution was prepared by adding 20 mg catalase (bovine liver, Sigma C1345-10G 2950 units/mg) to 10 ml of SDW. The dilutions of honey were made based on the results of two previous antibacterial screening studies [21,22]. To determine the phenol equivalence of each honey sample, 2% to 7% (w/v) phenol (crystallized, extra pure, Scharlau, FE0480, Spain) solutions were prepared and used as standards [25,26].

Each honey sample and controls were tested in triplicate by adding 140 μl to each well. Sterile distilled water, catalase solution, cefixime (5 μg) and ciprofloxacin (5 μg) were used as controls. The plates were incubated for 18 hours at 37°C after applications of samples and controls. The clear zones produced by each sample and control were measured in mm with digital calipers (Sylvac, Fowler, Ultra-Call11).

### Statistical analysis

For each experiment, three replicates were performed. The data were analyzed by using SPSS 15.0 after checking the assumption of normality. The arithmetic mean of observations and standard deviation of mean values were calculated. Statistical significance of the difference in the zone of inhibition among the different honey samples was determined with one way ANOVA (analysis of variance), and if found to be significant, a post hoc Tukey’s test was applied to evaluate the differences in the zone of inhibition between the honey samples. For all statistical tests, results were considered to be significant at p ≤ 0.05.

A standard reference curve was plotted for the % phenol solutions against the square mean diameter of the inhibition zone around each phenol concentration obtained from the agar well diffusion assay. The linear line of best fit was drawn and a regression equation generated using the Statistical Package for Social Sciences (SPSS 15.0). The phenol equivalence for each honey sample was calculated from the square mean diameter of the inhibition zone. The result was multiplied by a factor of 4.69 to account for the dilution and density of the honey assuming that the honey had a mean density of 1.35 mg/ml [21]. The antibacterial potential of each honey was then expressed as an equivalent phenol concentration (% w/v). Those honey samples which showed antibacterial activity equivalent to or greater than manuka honey were considered therapeutically active honeys.

## Results

### Phenol equivalence of honey samples

**Figure 1:**
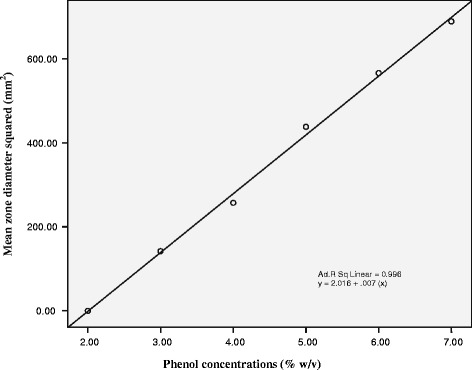
**Calibration curve for the phenol solutions used in the agar-well-diffusion assay of antibacterial potential against MDR-** ***Salmonella typhi*** **(UHS-16).**

None of the indigenous honey samples produced a zone of inhibition against MDR-*S. typhi* at 25% (w/v) dilution in sterile distilled water or in the presence of catalase solution. By comparison, manuka honey displayed a 12 mm inhibition zone at both 25% (w/v) dilution in sterile distilled water and in the presence of catalase. However, some indigenous honey samples showed a zone of inhibition at 50% (w/v) dilution in sterile distilled water and thus, their phenol equivalences were calculated (Table 3). The inhibition zones produced by different dilutions of phenol against MDR-*S. typhi* are shown in Table 3. Phenol at 2% (w/v) did not produce any zone of inhibition against MDR-*S. typhi*. However, the other phenol dilutions did exhibit a zone of inhibition. The inhibition zone tends to increase with increasing phenol concentration (Table 3). The calibration curve for the different phenol dilutions and the regression equation (y = 2.016 + .007 (x)) obtained are presented in Figure 1. The adjusted R square for the phenol standard curve was 0.996. The relationship between the phenol solutions and the zone of inhibition is linear over the entire range tested. Phenol equivalence was calculated for each indigenous honey sample from the phenol standard curve. Table 4 shows the antibacterial activity of each honey sample expressed in phenol equivalence (%w/v). The range of antibacterial activity of all indigenous honey samples tested in this study in terms of their phenol equivalences against MDR*-S. typhi* was obtained between 0 and 20%. This range was further divided into four groups: 0–5%, 6–10%, 11–15%, and 16–20%. The phenol equivalence obtained for manuka honey against MDR*-S. typhi* was 13%, thus classifying it as having significant activity (11–15%).

**Table 3:** **Zone of inhibition (mm) of phenol in triplicate against MDR-** ***Salmonella typhi*** **(UHS-16) by the agar well diffusion assay**

**Phenol concentration (w/v)**	**Mean zone size**	**Mean squared**
2%	0	0
3%	11.95 ± 0.08	142.80
4%	16.04 ± 0.02	257.28
5%	20.94 ± 0.07	438.48
6%	23.80 ± 0.74	566.44
7%	26.26 ± 0.28	689.58

**Table 4:** **Phenol equivalence of Pakistani honeys at 50**% **(w/v) dilution against MDR-** ***Salmonella typhi*** **expressed in Inhibine Antibacterial Units (IBU)**

**Honey samples (n)**	**Phenol equivalence % (w/v)/ IBU**			
	**0--5**	**6--10**	**11--15**	**16--20**
Manuka (Standard)	0	0	1	0
Acacia (15)	10	0	3	2
Beri (29)	7	0	12	10
Bhaiker (5)	4	0	1	0
Black locust (3)	3	0	0	0
Black seed (5)	1	0	4	0
Citrus (12)	11	0	1	0
Clover (4)	2	0	1	1
Date palm (1)	0	0	1	0
Eucalyptus (1)	0	0	0	1
Garanda (4)	0	0	3	1
Honey dew (1)	0	0	0	1
Lavender (1)	1	0	0	0
Litchi (2)	2	0	0	0
Loquat (1)	1	0	0	0
Mustard (4)	3	0	1	0
Russian olive (5)	4	0	1	0
Shain (1)	0	0	0	1
Sun flower (5)	2	0	2	1
Walati kikher (1)	0	0	0	1
**Total**	**51**	**0**	**30**	**19**

Key: 0–5, insignificant activity; 6–10, low activity; 11–15, significant activity; 16–20, high activity.

### Rating the antibacterial activity of the Pakistani honey samples

A total of 51% of honey samples had phenol equivalence between 0 and 5% (insignificant activity group) against MDR-*S. typhi* at 50% (w/v) dilution in SDW (Table 4). A total of 30% of the honey samples demonstrated antibacterial activity between the ranges of 11–15% phenol (significant activity group), similar to that of manuka honey (Table 4). Out of the four tested, three garanda honey samples displayed antibacterial activity between the ranges of 11–15%, similar to that of manuka honey. One garanda honey sample showed antibacterial activity between 16 and 20% (high activity group), higher than that of manuka honey. A total of 12 beri honey samples displayed significant antibacterial activity (11–15% of phenol), similar to that of manuka honey. Moreover, ten beri honey samples displayed antibacterial activity greater than that of manuka honey. Out of five black seed honey samples, four were in the significant group (11–15% of phenol), similar to that of manuka honey (Table 4). Overall, 19 honey samples displayed higher antibacterial activity (16–20% of phenol) than that of manuka honey, including beri (n = 10), acacia (n = 2), clover (n = 1), eucalyptus (n = 1), garanda (n = 1), sunflower (n = 1), honey dew (n = 1), walati kikher (n = 1) and shain (n = 1) (Table 4).

### Mean and standard deviation of the antibacterial activity of different honey varieties

Mean and standard deviation of the antibacterial activity of the honey groups comprising more than one sample are shown in Table 5. Overall, garanda honey exhibited the highest antibacterial activity (15.25 ± 0.50), followed by beri honey (11.76 ± 6.8) and black seed honey (11.60 ± 6.5). Robinia and litchi honey did not show any activity in this study. One way analysis of variance (ANOVA) found a significant difference (p = 0.0001) in the size of the inhibition zone among the different honey groups demonstrating the variation in the potency of antibacterial activity among different honey groups.

**Table 5:** **Mean and standard deviation of the antibacterial activity of different honey groups according to floral source (expressed as phenol equivalence % (w/v) against MDR-** ***Salmonella typhi*** **(UHS-16) and 90**% **Confidence Intervals for the means**

**Honey samples (n)**	**Mean ± SD**	**95%** CI* **for mean**	
		**Lower limit**	**Upper limit**
Beri (29)	11.76 ± 6.8	9.16	14.36
Acacia (15)	05.13 ± 7.5	0.94	9.33
Citrus (12)	01.25 ± 4.3	−1.50	4.00
Black seed (5)	11.60 ± 6.5	3.52	19.68
Bhaiker (5)	02.80 ± 6.2	−4.97	10.57
Sunflower (5)	09.00 ± 8.2	−1.24	19.24
Russian olive (5)	02.80 ± 6.2	−4.97	10.57
Garanda (4)	15.25 ± 50	14.45	16.05
Mustard (4)	03.50 ± 7.0	−7.64	14.64
Clover (4)	07.50 ± 8.6	−6.34	21.34
Robinia (3)	0	0	0
Litchi (2)	0	0	0

*CI, confidence interval.

## Discussion

### Hydrogen per-oxide related antibacterial activity

Out of 100 honey samples, 19% displayed higher antibacterial activity (16–20% of phenol) at 50% dilution compared with that of manuka (Table 4). These included beri, acacia, clover, eucalyptus, garanda, sunflower, honey dew, walati kikher and shain honey (Table 4). However, garanda honey, beri honey and black seed honey showed higher mean antibacterial activity (Table 5). This indicated the occurrence of a high level of hydrogen peroxide related antibacterial activity in these indigenous honey samples. Previously, buckwheat honey, ulmo honey and jarrah honey were found to contain high levels of hydrogen peroxide activity [25,27,28]. Hydrogen peroxide, present in most honey at variable concentrations, is generally considered to be the main contributor to the antibacterial activity of honey [27,29]. However, the concentration of H_2_O_2_ in honey is approximately 400–4000 times lower than that required for bacteriolysis [9] and hydrogen peroxide alone is less bacteriostatic than the equivalent amount present in honey [30]. It has been shown that the antibacterial activity of honey can be augmented by polyphenols and a synergistic interaction was identified between H_2_O_2_, polyphenols and transition metals [31]. These findings further strengthen the notion that hydrogen peroxide is one of main compounds involved in the antibacterial activity of honey. Unlike hydrogen peroxide induced cytotoxicity when tested in isolation, honey has no tissue damaging effects because it generates optimal levels of hydrogen peroxide to the applied areas [32]. The antibacterial effects of •OH are more potent as compared to H_2_O_2_ because the former cannot be neutralized by any enzyme [33]. Recently, it has been revealed by a 3′-(*p*-aminophenyl) fluorescein (APF) •OH trap technique that the oxidative stress induced by honey on bacterial cells is not related to molecular H_2_O_2_ but rather the presence of the •OH radical generated from honey H_2_O_2_. A recent study revealed that •OH inhibited the growth of both multi-drug resistant (MRSA and VRE) and standard bacteria (*E. coli* and *B. subtilis*) [34]. Unlike H_2_O_2_ and methylglyoxal, which are inactivated by catalase and gastric enzymes respectively, •OH is a stable substance which is not inactivated by any endogenous or exogenous enzyme [33,35]. These findings open new avenues for future research into the induction of •OH containing honeys as a potential therapeutic agent for wound and systemic infections. The presence of •OH produced by honey could be a valuable marker for determination of the antibacterial efficacy of honeys for clinical applications [34]. Currently, the non-peroxide activity of manuka honey is given more importance clinically than its peroxide based activity, which is measured in term of unique manuka factor [21]. The difference in clinical efficacy of UMF honey like manuka and high hydrogen peroxide containing honey like buckwheat, ulmo and jarrah [25,27,28] to treat wound infections has not been tested yet.

### Absence of antibacterial activity of honey samples

A total of 51% of honey samples did not exhibit any antibacterial activity against MDR-*S. typhi* at any dilution (Table 4). Out of 12, only one sample of citrus honey showed antibacterial activity. Similarly, most of the acacia, bhaiker, Russian olive and mustard honey samples fail to exhibit any antibacterial activity against MDR-*S. typhi*. Moreover, all samples of robinia, pseudoacacia and litchi honey were unable to produce antibacterial activity against MDR-*S. typhi*. In a previous study where 340 honeys were screened, 68.5% did not demonstrate antibacterial activity against *S. aureus* (ATCC 25923) [22]. A study of 345 honey samples showed that 36% had antibacterial activity against *S. aureus* (ATCC 25923) at concentrations below the level of detection [21]. However, direct comparison of these results to previous studies is often difficult because of differences in test organisms. Lack of antibacterial activity of honey samples might be as a result of improper processing or handling of honey. In Pakistan, the overwhelming majority of people believe that any honey that granulates is altered or synthetic. Therefore, the majority of bee keepers and packers heat the honey in order to prevent crystallization/granulation. However, the majority of honeys produced in the world tend to granulate naturally [36]. Heating adversely affects the quality of the honey by destroying a number of valuable enzymes, including oxidase which is responsible for the production of hydrogen peroxide in honey [37]. Therefore, it is important that honey should not be heated during processing and be stored in brown glass containers like other medical products [37]. The production of clinically valuable honeys requires the honey to be collected, processed and stored under standard conditions. This demands the identification of the appropriate floral sources, and the development of procedures for harvesting and handling [22].

### Variations in the level of antibacterial activity of honey samples

Like the previously reported preliminary studies, these results also demonstrate that the antibacterial activity of honey varies tremendously among different honey samples [21,38,39] (Table 4). A high level of antibacterial activity against MDR-*S. typhi* was found to be associated with garanda honey, beri honey and black seed honey. Conversely, robinia honey, litchi honey, loquat honey, black locust honey and lavender honey did not show any detectable antibacterial activity in this assay (Table 4). A profound degree of disparity in the antibacterial activity of honey from the same floral source but from different geographical locations was also observed in this study as documented in other studies [21,39,40] (Table 4). For example, beri honey showed a high degree of variation (0–18% of phenol equivalence) among the samples collected from different geographical areas of Pakistan (Table 4). A high degree of activity was observed for beri honey samples collected from the Karak district of Pakistan. Generally low activity was detected in beri samples collected from the Attock district. The difference in antibacterial activities within the same floral source could be related to soil composition, influence of climate, processing of honey and concentration of propolis [41]. Previous reports also mentioned the marked degree of variation in the antibacterial potential of 50 manuka honey samples (16.2 ± 10.5), collected from different geographical locations of New Zealand. Non-peroxide activity was attributed to only 38% of the samples and many did not show any detectable activity [21]. This result also confirms that the antibacterial activity is not associated with each and every honey sample.

### Non-peroxide antibacterial activity of honey samples

None of the indigenous honey samples exhibited non-peroxide activity, whereas manuka honey (UMF-21) showed non-peroxide activity against MDR-*S. typhi*. The absence of non-peroxide based antibacterial activity of the indigenous honey samples could be attributed to the lack of sensitivity of the agar well diffusion assay. The indigenous honey samples may contain large antibacterial compounds which may not diffuse out from the well and thus be unable to produce a zone of inhibition. For instance, Polymyxin, a large compound, is unable to diffuse in a disk diffusion test, hence MIC type assays, like the agar dilution assay or broth dilution assay are recommended for determining its efficacy [42]. Therefore a more sensitive assay may be necessary for determining the antibacterial activity of honey. However, manuka honey displayed a 12 mm inhibition zone against MDR-*S. typhi* after the addition of catalase solution at 25% dilution. Generally these results are in agreement with previous reports, however, previous studies evaluated manuka honey against *Staphylococcus aureus* (ATCC 25923) and showed that it exhibited an inhibition zone of 14 mm against *Staphylococcus aureus* (ATCC 25923) [21,22]. The difference in the size of the inhibition zone is most likely because of the difference in test organisms. It has been shown in previous studies that *S. aureus* is more sensitive to honey than *S. typhi* [43].

### Phenol equivalence of honey samples

The phenol equivalence of each indigenous honey sample against MDR-*S. typhi* was calculated from the phenol standard curve (Figure 1). The phenol equivalence obtained for each honey sample is the measure of the total antibacterial activity (peroxide and non-peroxide) of honey against *S. typhi* taking into account that the honey samples were diluted 50% in sterile distilled water without catalase. The total activity of the honey samples expressed in phenol equivalence is quoted in Inhibine Antibacterial Units (IBU) in this study. However, the phenol equivalence of each honey sample in terms of its non-peroxide activity was not determined because none of the indigenous honey samples showed antibacterial activity in catalase solution. The inhibine consists of hydrogen peroxide, flavonoids, phenolic acids and several other unidentified substances present in honey [43]. Thus, the word inhibine represents the total antibacterial activity of honey, and therefore IBU represents a measure of the total antibacterial activity of honey samples expressed in phenol equivalence. IBUs were grouped into four ranges: 0–5, 6–10, 11–15 and 16–20 (Table 4). The manuka honey used in this study is a standardized product with 21 UMF and approved by the FDA as therapeutic agent for wound care [44]. Therefore, those honey samples which displayed antibacterial activity greater than manuka honey may be useful in the clinical conditions where a higher level of hydrogen peroxide related antibacterial activity is required.

### Potential delivery methods of honey for salmonella typhi infection

One important issue to consider when using honey to treat typhoid fever is how to achieve a honey concentration in target organs like spleen, liver, lymphoid tissue of the small intestine and blood at sufficient concentrations to be both active and bactericidal for multi-drug resistant *S. typhi*. To achieve the necessary concentrations for treatment of *S. typhi*, honey could be delivered orally or intravenously. Honey has been used as a food since antiquity, present almost everywhere and can be given safely by the oral route. However, the disadvantage of the oral route would be the dilution of honey by large amounts of body fluid e.g. saliva, gastric juice, intestinal fluid and water from food and drink [45]. Therefore, it is uncertain how much honey would be able to reach the blood, spleen, liver and other locations where *S. typhi* may colonize. Second, H_2_O_2_ activity could be neutralized by catalase present in tissues and blood. Hence, non-H_2_O_2_ containing honey like manuka, which contains methylglyoxal, may be more suited to treat typhoid fever. However, it has been shown that methylglyoxal present in manuka honey is readily digested and inactivated in the gastrointestinal tract thus the concentration of honey achievable at site of infection would be much lower than the concentration required to be bactericidal against *S. typhi* [35]. Therefore, use of the oral route of honey delivery for eradication of *S. typhi* might not be useful. Nevertheless, it is worth mentioning that recently it has been revealed that polyphenols present in honey generate •OH from H_2_O_2_ via Fenton activity in the presence of Cu(I) or Fe(II) and the antibacterial effects of •OH are more potent compared with H_2_O_2_ because the former cannot be neutralized by any enzyme [34]. Therefore, the honeys which contain higher amounts of polyphenols and hydrogen peroxide like buckwheat honey may produce more •OH and thus, be more effective *in vivo* against *S. typhi*. More recently, it has been shown that the stability and bioactivity of methylglyoxal can be increased by coupling it with cyclodextrins [35]. The other possible means of honey administration is the intravenous route which may allow a concentration of honey greater than the MIC to be achieved in the serum of a patient with typhoid fever. The usefulness and safety of intravenous honey (*i.v.*) has been shown in healthy sheep where slow *i.v.* infusion or rapid *i.v*. injection of honey at different concentrations (5% and 40% in normal saline or sterile distilled water) was safe, could lower blood sugar and improved renal, hepatic, and bone marrow functions and the lipid profile [46]. However, in order to confirm the efficacy of oral or intravenous honey against typhoid fever, pharmacodynamic studies in mouse typhoid model are ongoing in our laboratory.

## Conclusions

The results in the present study revealed that Pakistani honeys as well as manuka honey has antibacterial activity against MDR-*Salmonella typhi.* The results also demonstrated the presence of a higher level of hydrogen peroxide related antibacterial activity in some indigenous honey samples (19%) against MDR-*Salmonella typhi* compared with manuka (UMF-21) honey. Therefore, these honey samples could be utilized in the clinic where higher H_2_O_2_ related antibacterial activity is required. However, only manuka honey displayed non-peroxide antibacterial activity in this study while the indigenous honey samples did not. Therefore, manuka honey warrants further evaluation in a suitable typhoid animal model for evaluation of its utility in the prevention and treatment of typhoid fever.

## Abbreviations

API: Analytical profile index
ATCC: American type culture collection
IBU: Inhibine antibacterial unit
MDR: Multi drug resistance
UMF: Unique manuka factor
